# Replacement of Marine Fish Oil with *de novo* Omega-3 Oils from Transgenic *Camelina sativa* in Feeds for Gilthead Sea Bream (*Sparus aurata* L.)

**DOI:** 10.1007/s11745-016-4191-4

**Published:** 2016-09-02

**Authors:** Mónica B. Betancor, M. Sprague, D. Montero, S. Usher, O. Sayanova, P. J. Campbell, J. A. Napier, M. J. Caballero, M. Izquierdo, D. R. Tocher

**Affiliations:** 1 Faculty of Natural Sciences, Institute of Aquaculture, University of Stirling, Stirling, FK9 4LA UK; 2 Grupo de Investigación en Acuicultura (GIA), Instituto Universitario Ecoaqua, Universidad de Las Palmas de Gran Canaria, Ctra. Taliarte s/n, 35214 Telde, Las Palmas, Canary Islands Spain; 3 Department of Biological Chemistry and Crop Protection, Rothamsted Research, Harpenden, AL5 2JQ UK; 4 Biomar Ltd., North Shore Road, Grangemouth, FK3 8UL UK

**Keywords:** Sea bream, Genetically modified, Camelina, Sustainable feeds

## Abstract

Omega-3 (n-3) long-chain polyunsaturated fatty acids (LC-PUFA) are essential components of the diet of all vertebrates. The major dietary source of n-3 LC-PUFA for humans has been fish and seafood but, paradoxically, farmed fish are also reliant on marine fisheries for fish meal and fish oil (FO), traditionally major ingredients of aquafeeds. Currently, the only sustainable alternatives to FO are vegetable oils, which are rich in C_18_ PUFA, but devoid of the eicosapentaenoic (EPA) and docosahexaenoic acids (DHA) abundant in FO. Two new n-3 LC-PUFA sources obtained from genetically modified (GM) *Camelina sativa* containing either EPA alone (ECO) or EPA and DHA (DCO) were compared to FO and wild-type camelina oil (WCO) in juvenile sea bream. Neither ECO nor DCO had any detrimental effects on fish performance, although final weight of ECO-fed fish (117 g) was slightly lower than that of FO- and DCO-fed fish (130 and 127 g, respectively). Inclusion of the GM-derived oils enhanced the n-3 LC-PUFA content in fish tissues compared to WCO, although limited biosynthesis was observed indicating accumulation of dietary fatty acids. The expression of genes involved in several lipid metabolic processes, as well as fish health and immune response, in both liver and anterior intestine were altered in fish fed the GM-derived oils. This showed a similar pattern to that observed in WCO-fed fish reflecting the hybrid fatty acid profile of the new oils. Overall the data indicated that the GM-derived oils could be suitable alternatives to dietary FO in sea bream.

## Introduction

Fish is considered as the main source of the beneficial omega-3 (n-3) long-chain polyunsaturated fatty acids (LC-PUFA) eicosapentaenoic and docosahexaenoic acids, EPA (20:5n-3) and DHA (22:6n-3), respectively. These LC-PUFA play important roles in neural development, immune and inflammatory responses as well as having beneficial effects in certain pathologies such as those affecting the cardiovascular and neurological systems or some types of cancers [1–5]. According to estimations of the International Society for the Study of Fatty acids and Lipids (ISSFAL), a daily intake of 500 mg of EPA and DHA is recommended for optimum cardiovascular health [6]. In a world where the global population is expected to grow to reach 9.6 billion people by 2050 [7], more than 1.7 million metric tonnes of EPA + DHA would be necessary to cover annual human requirements. This quantity is not met by the actual total global supply of n-3 LC-PUFA and thus there is a large gap between supply and demand [8, 9].

Farmed fish and seafood accounted for 44.1 % of total production (including for non-food uses) from capture fisheries and aquaculture in 2014, up from 42.1 % in 2012 and 31.1 % in 2004 [7]. Although fish farming contributes to the global n-3 LC-PUFA production in order to meet human dietary requirements, the marine ingredients, fish meal and oil (FM and FO, respectively) are still, almost exclusively, the only raw materials in aquafeeds that, in turn, can supply n-3 LC-PUFA to farmed fish. The use of high levels of FM and FO to maintain n-3 LC-PUFA levels in farmed fish is not a sustainable practice as they are finite (on an annual basis) and limited resources [9]. Sustainable alternatives to FO used at present are vegetable oils (VO), which are rich in C_18_ but lack n-3 LC-PUFA, which in turn reduces the proportions of EPA and DHA in farmed fish, and does not significantly increase global production of n-3 LC-PUFA [9]. Obtaining alternative sources of n-3 LC-PUFA from other marine organisms such as microalgae or zooplankton (krill or copepods) poses significant technological and economic challenges and there are no currently feasible alternatives for mass supply [9]. Therefore, it is clear that completely new, *de novo* sources of EPA and DHA are required. In this respect, metabolic engineering of oilseed crops such as *Camelina sativa* or false flaxseed provides a currently viable option to deliver n-3 LC-PUFA in the place of fish oil [10–12]. By the insertion of cassettes containing five or seven fatty acyl desaturase and elongase genes from several algae species, genetically modified (GM) Camelina is capable of producing EPA or both EPA and DHA in their seeds [10]. Recent studies have successfully demonstrated the feasibility of using both the high-EPA and EPA + DHA oils as substitutes for FO in feeds for post-smolt Atlantic salmon (*Salmo salar*) without compromising fish growth or health [13–15].

Total replacement of FO in salmonids has been shown to be feasible without compromising fish performance, although a reduction in tissue n-3 LC-PUFA was observed due to reduced dietary intake and limited biosynthesis [16–18]. In contrast, total substitution of FO by VO in gilthead sea bream (*Sparus aurata*) feeds significantly reduced fish performance [19] and greatly altered fatty acid profile of bream tissues [20] given their incomplete LC-PUFA biosynthesis pathway [21, 22]. In this context, the aim of the present study was to evaluate the new GM Camelina-derived oils that contain features of both marine fish (n-3 LC-PUFA) and terrestrial plant (high levels of C_18_ fatty acids) oils as substitutes for FO in feeds for a marine teleost species that have a very limited LC-PUFA biosynthesis capacity from C_18_ PUFA [22]. The specific objectives were to evaluate the efficacy of the high-EPA and EPA + DHA oils as replacements for dietary FO in feeds for gilthead sea bream juveniles in terms of growth, feed efficiency, health and welfare, and nutritional quality of the fish focussing particularly on tissue levels of EPA and DHA. Additionally, assessment of the expression of genes of lipid metabolism as well as molecular markers of health and immune function was also performed.

## Materials and Methods

### Diets and Feeding Trial

Four isonitrogenous and isoenergetic diets were formulated to contain 50 g/kg crude protein and 11 g/kg crude lipid, and manufactured at BioMar Tech-Centre (Brande, Denmark). The four feeds were produced by vacuum coating identical dry basal extruded pellets with either fish oil (FO), wild-type Camelina oil (WCO), EPA-Camelina oil (ECO) or EPA + DHA-Camelina oil (DCO) and were named according to the oils used (Table 1). ECO and DCO oils were produced as previously described [10, 13, 15]. In line with commercial practice, non-defatted fishmeal was employed as the major protein source to ensure EFA requirements were met [23], and yttrium oxide was added (0.5 g/kg) as an inert marker for calculation of lipid and fatty acid digestibility.

**Table 1:** Formulations, proximate and fatty acid compositions (% of fatty acids) of the experimental feeds

	FO	WCO	ECO	DCO
Feed ingredients (%)				
Fish meal, NA LT 70	24.43	24.43	24.43	24.43
Fish meal, SA 68 Superprime	24.43	24.43	24.43	24.43
Soy protein concentrate (60 %)	9.79	9.79	9.79	9.79
Maize gluten	14.66	14.66	14.66	14.66
Wheat	14.66	14.66	14.66	14.66
Fish oil^a^	11.53	–	–	–
Wild-type Camelina oil^a^	–	11.53	–	–
EPA-Camelina oil^a^	–	–	11.53	–
EPA + DHA-Camelina oil^a^	–	–	–	11.53
Vitamins/minerals	0.45	0.45	0.45	0.45
Yttrium oxide	0.05	0.05	0.05	0.05
Analysed composition				
Dry matter (%)	93.78	93.57	94.05	93.94
Crude protein (%)	50.00	50.72	50.23	50.53
Crude lipid (%)	18.47	18.27	17.90	17.93
Ash	8.37	8.33	8.45	8.15
Fatty acid composition (mol%)				
∑ saturated^b^	32.60	15.89	18.84	19.71
∑ monounsaturated^c^	27.55	31.93	21.25	22.23
18:2n-6	5.61	18.25	21.90	19.72
20:2n-6	0.15	1.31	1.46	0.78
20:3n-6	0.13	n.d	1.17	0.82
20:4n-6	1.09	0.22	2.44	1.53
22:4n-6	0.07	n.d.	n.d.	n.d.
∑ n-6 PUFA^d^	7.56	19.85	28.16	26.56
18:3n-3	1.03	23.78	8.68	10.96
20:3n-3	0.06	0.89	0.80	0.52
20:4n-3	0.55	0.14	2.15	1.66
20:5n-3	13.15	2.31	13.53	6.24
22:5n-3	1.39	0.25	0.78	1.21
22:6n-3	9.67	3.91	4.20	6.88
∑ n-3 PUFA	27.86	31.84	31.25	30.99
∑ PUFA^e^	39.85	52.18	59.91	58.06
Total n-3 LC-PUFA	24.75	6.61	20.66	15.99

*n.d.* not detected, *WCO* wild-type Camelina oil feed

^a^Fatty acid compositions of the oils were provided previously [13, 15]

^b^Includes 15:0, 22:0 and 24:0

^c^Includes 16:1n-9, 20:1n-11, 20:1n-7, 22:1n-9 and 24:1n-9

^d^Includes 22:4n-6 and 22:5n-6

^e^Includes C_16_ PUFA. DCO, feed containing EPA + DHA oil from transgenic Camelina; ECO, feed containing high-EPA oil from transgenic Camelina; FO, fish oil feed; LC- PUFA, long-chain polyunsaturated fatty acids (sum of 20:4n-3, 20:5n-3 22:5n-3 and 22:6n-3)

A total of 420 juvenile gilthead sea bream with an average body weight of 55.5 ± 0.4 g (mean ± SD) were distributed into 12 seawater tanks (35 per tank) and fed one of the four experimental feeds in triplicate for 11 weeks. The experimental system comprised 500 L tanks supplied by flow-through seawater (9 L/min) at ambient temperature that averaged 20.3 ± 0.6 °C. Experimental feeds were delivered until apparent satiation by hand-feeding three times a day with uneaten feed collected 30 min later in order to determine accurate feed efficiency. Collected feed was placed in an oven (110 °C) until constant weight was achieved in order to calculate feed intake based on initial moisture content. All procedures were conducted in accordance with the regulations set forward by the Spanish RD 53/2013 (BOE 8th February 2013) and the Directive 2010/63/EU of the European Parliament and of the Council of 22 September 2010 on the protection of animals used for scientific purposes. The experiment was subjected to ethical review by the Animal Welfare and Bioethical Committee at the University of Las Palmas de Gran Canaria (Ref 007/202 CEBA ULPGC).

### Sample Collection and Digestibility

After 11 weeks of feeding, fish were not fed for 48 h prior to being sampled. All fish were anaesthetised with clove oil and weighed and their length measured. Ten fish for biometric measurements (hepato-somatic and viscero-somatic indices) and tissue analyses were killed by overdose with clove oil. Samples of anterior intestine, liver, gills, flesh and brain from 3 fish per tank were immediately frozen and stored at −70 °C prior to total lipid extraction and fatty acid analyses. Further samples of liver and anterior intestine were collected from six fish per treatment (two per tank) and stabilised in RNAlater^®^ (Sigma, Poole, UK) prior to RNA extraction. All remaining fish were fed for a further week prior to faeces being collected according to the method described by [24]. Briefly, fish were killed by overdose with clove oil 7 h after being fed and faecal samples collected after dissecting the rectum of the fish. Faecal samples were pooled by tank and stored −20 °C prior to lipid and fatty acid analysis. The apparent digestibility coefficient (ADC) of lipid and selected fatty acids was calculated as: 100 − [100 × (Y_2_O_3_ concentration in feed/Y_2_O_3_ concentration in faeces) × (lipid or fatty acid concentration in faeces/lipid or fatty acid concentration in feed)]. The concentration of individual fatty acids in diets and faeces were calculated based on the relative proportion of each fatty acid compared with a known amount of the internal standard (17:0) added and the total lipid content determined in the samples. Yttrium was estimated after acid digestion of the samples via Inductively Coupled Plasma Mass Spectrometry (Thermo Scientific, XSeries2 ICP-MS, USA) using argon and hydrogen as carrier gas.

### Proximate Composition

Diets and whole fish were ground before determination of proximate composition according to standard procedures [25]. Fish were pooled per tank (*n* = 3) and freeze-dried until further analysis whereas three technical replicates of feeds (single batch production) were analysed. Moisture contents were obtained after drying in an oven at 110 °C for 24 h and ash content determined after incineration at 600 °C for 16 h. Crude protein was measured by determining nitrogen content (N × 6.25) using automated Kjeldahl analysis (Tecator Kjeltec Auto 1030 analyser, Foss, Warrington, UK) and crude lipid content determined gravimetrically after Soxhlet lipid extraction (Tecator Soxtec system 2050 Auto Extraction apparatus).

### Faeces and Tissue Lipid Content and Fatty Acid Composition

Samples of faeces, muscle (flesh), liver, gills, anterior intestine and brain from three fish per tank were prepared as pooled homogenates (*n* = 3 per treatment) and total lipid extracted by homogenising in chloroform/methanol (2:1, v/v) using an Ultra-Turrax tissue disrupter (Fisher Scientific, Loughborough, UK), with content determined gravimetrically [26]. Fatty acid methyl esters (FAME) were prepared from total lipid by acid-catalysed transesterification at 50 °C for 16 h [27], and FAME extracted and purified as described previously [28]. FAME were separated and quantified by gas-liquid chromatography using a Fisons GC-8160 (Thermo Scientific, Milan, Italy) equipped with a 30 m × 0.32 mm i.d. × 0.25 μm ZB-wax column (Phenomenex, Cheshire, UK), on-column injector and a flame ionisation detector. Data were collected and processed using Chromcard for Windows (version 2.01; Thermoquest Italia S.p.A., Milan, Italy). Individual FAME were identified by comparison to known standards and published data [28] and results expressed as mole percentage.

### Histological Analysis

Samples of liver and intestine from 2 fish per tank (*n* = 6 per treatment) were fixed in 4 % buffered formalin dehydrated through graded alcohol, then xylene, and finally embedded in paraffin wax. The paraffin blocks were sectioned at 3 μm and stained with haematoxylin and eosin [29] before blind examination under a light microscope. Stained sections of liver were assessed for cytoplasmic lipid vacuolization and peripancreatic fat infiltration using a four graded examination scheme: 0, not observed; 1, few; 2, medium; 3, severe. Posterior intestine sections were examined for integrity of the intestinal mucosa and the presence of any inflammatory response.

### RNA Extraction

Liver and anterior intestine from six individual fish per dietary treatment were homogenised in 1 ml of TriReagent^®^ (Sigma-Aldrich, Dorset, UK) RNA extraction buffer using a bead tissue disruptor (Bio Spec, Bartlesville, Oklahoma, USA). Total RNA was isolated following the manufacturer’s instructions and quantity and quality determined by spectrophotometry using a Nanodrop ND-1000 (Labtech Int., East Sussex, UK) and electrophoresis using 250 ng of total RNA in a 1 % agarose gel. cDNA was synthesised using 2 μg of total RNA and random primers in 20 μl reactions and the High capacity reverse transcription kit without RNase inhibiter according to the manufacturer’s protocol (Applied Biosystems, Warrington, UK). The resulting cDNA was diluted 20-fold with milliQ water. Expression of genes of interest was determined by quantitative PCR (qPCR) from fish fed all diets (Table 2). Results were normalised using reference genes, *elongation factor 1α* (*elf1α*) and *beta-actin* (*actb*), as their expression did not vary among treatments. The efficiency of the primers for each gene was previously evaluated by serial dilutions to ensure that it was close to 100 %. qPCR was performed using a Biometra TOptical Thermocycler (Analytik Jena, Goettingen, Germany) in 96-well plates in duplicate 20-μl reaction volumes containing 10 μl of Luminaris Color HiGreen qPCR Master Mix (Thermo Scientific, Hemel Hempstead, UK), 1 μl of the primer corresponding to the analysed gene (10 pmol), 3 μl of molecular biology grade water and 5 μl of cDNA, with the exception of the reference genes, which were determined using 2 μl of cDNA. In addition amplifications were carried out with a systematic negative control (NTC-non template control) containing no cDNA. Standard amplification parameters contained a UDG pre-treatment at 50 °C for 2 min, an initial denaturation step at 95 °C for 10 min, followed by 35 cycles: 15 s at 95 °C, 30 s at 60 °C and 30 s at 72 °C.

**Table 2:** Details of primer used for qPCR or PCR analysis

Aim	Transcript	Primer sequence (5′ → 3′)	Amplicon (bp)	*T*a (°C)	Accession no.
*qPCR*	*fads2*	F: GCAGGCGGAGAGCGACGGTCTGTTCC	72	60	AY055749
		R: AGCAGGATGTGACCCAGGTGGAGGCAGAAG			
	*elovl4*	F: CGGTGGCAATCATCTTCC	79	60	JX975701
		R: TCAACTGGCTGTCTGTGT			
	*elovl5*	F: CCTCCTGGTGCTCTACAAT	112	60	AY660879
		R: GTGAGTGTCCTGGCAGTA			
	*lpcat1*	F: CGTGATAGCCTTATCTGTCGTATGC	90	60	JQ390612
		R: CCGTCCTCCTCTGCCTCAA			
	*FABP2*	F: CGAGCACATTCCGCACCAAAG	93	60	AM957164
		R: CCCACGCACCCGAGACTTC			
	*hl*	F: TTGTAGAAGGTGAGGAAAACTG	131	60	EU254479
		R: GCTCTCCATCAGACCATCC			
	*lpl*	F: CGTTGCCAAGTTTGTGACCTG	192	60	AY495672
		R: AGGGTGTTCTGGTTGTCTGC			
	*cpt1a*	F: GTGCCTTCGTTCGTTCCATGATC	82	60	JQ308822
		R: TGATGCTTATCTGCTGCCTGTTTG			
	*cpt1b*	F: CCACCAGCCAGACTCCACAG	78	60	DQ866821
		R: CACCACCAGCACCCACATATTTAG			
	*srebp1*	F: AGGGCTGACCACAACGTCTCCTCTCC	77	60	JQ277709
		R: GCTGTACGTGGGATGTGATGGTTTGGG			
	*PPARα*	F: TCTCTTCAGCCCACCATCCC	116	60	AY590299
		R: ATCCCAGCGTGTCGTCTCC			
	*PPARγ*	F: CGCCGTGGACCTGTCAGAGC	103	60	AY590304
		R: GGAATGGATGGAGGAGGAGGAGATGG			
	*casp3*	F: CCAGTCAGTCGAGCAGATGA	113	60	EU722334
		R: GAACACACCCTCGTCTCCAT			
	*pcna*	F: GATGTGGAGCAGCTGGGTAT	205	60	FG263675
		R: TGTCTACGTTGCTGGTCTGG			
	*il8*	F: CAGCAGAGTCTTCATCGTCACTATTG	66	60	JX976619
		R: AGGCTCGCTTCACTGATGG			
	*actb*	F: TCCTGCGGAATCCATGAGA	50	60	X89920
		R: GACGTCGCACTTCATGATGCT			
	*ef1a*	F: ACGTGTCCGTCAAGGAAATC	109	60	AF184170
		R: GGGTGGTTCAGGATGATGAC			
*PCR*	*nptII*	F: CTCACCTTGCTCCTGCCGAGA	215	60	KJ081792.1
		R: CGCCTTGAGCCTGGCGAACAG			
	*cytb*	F: CCGCTTCTTTGCCTTCCATT	219	57	NC_024236.1
		R: AGATTAGGGGCGAATAGGGC			

*fads2* fatty acid desaturase 2, *elovl4* fatty acid elongase 4, *elovl5* fatty acid elongase 5, *lpcat1* lysophosphatidylcholine acyltransferase 1, *FABP2* fatty acid binding protein 2, *hl* hepatic lipase, *lpl* lipoprotein lipase, *cpt1a* carnitine palmitoyltransferase 1A liver isoform, *cpt1b* Carnitine palmitoyltransferase 1b muscle isoform, *srebp1* sterol regulatory element binding protein 1, *PPARα* peroxisome proliferator-activated receptor α, *PPARγ* peroxisome proliferator-activated receptor γ, *casp3* caspase 3, *pcna* proliferating cell nuclear antigen, *il8* interleukin 8, *actb* β-actin, *ef1α* elongation factor 1, *nptII* neomycin phosphotransferase II, *cytb* cytochrome b

### Tracking of the *nptII* Gene in Gilthead Sea Bream Liver, Intestine and Muscle

Genomic DNA was extracted from fish flesh, pyloric caeca and liver using REALPURE extraction kit (Valencia, Spain) according to the manufacturer’s instructions. Briefly, tissue samples were incubated in 300 μl of lysis solution overnight at 55 °C with 3 μl of Proteinase K. Following the incubation, samples were cooled down and RNase treatment performed (37 °C for 60 min). After protein precipitation, DNA was precipitated by adding 600 μl of isopropanol and hydrated with 5 mM Tris. Total DNA was quantified by spectrophotometry and quality determined by electrophoresis as described above. Two primers pairs targeting an endogenous sea bream gene (cytochrome b; *cytb*) and a transgene marker for ECO – plants (Kanamycin resistance gene, *nptII*) were used (Table 2). Fifty ng of extracted DNA was used in PCR amplifications that were performed in a final volume of 10 μl, containing 5 μl of MyTaq™ HS Mix (Bioline, London, UK). Each set of PCR included a positive control (DNA from genetically modified-Camelina) and a non-template control (NTC).

### Statistical Analysis

All data are mean ± SE (*n* = 3) unless otherwise specified. Percentage data were subjected to arcs in square-root transformation prior to statistical analyses. Data were tested for normality and homogeneity of variances with Levene’s test prior to one-way analysis of variance followed by a Tukey–Kramer HSD multiple comparisons of means. A non-metric multidimensional scaling plot (NMDS) was performed in order to separate the fatty acid profile of the five evaluated tissues. Stress values <0.05 indicated an excellent representation of the clusters and <0.1 and <0.2 indicated good and potentially useful plots respectively. All statistical analyses were performed using SPSS software (IBM SPSS Statistics 19; SPSS Inc., Chicago, IL, USA), excepting the NMDS analysis (PAST) [30].

## Results

### Fish Performance

Sea bream fed all the experimental diets more than doubled in weight after 11 weeks feeding, and no mortalities were recorded (Table 3). Fish fed ECO displayed the lowest final weight and total length among the dietary treatments, although not different to fish fed WCO, with highest growth achieved in fish fed the FO and DCO feeds (Table 3). Final weights reflected feed intake, which was highest in fish fed FO and DCO, lowest in fish fed ECO and intermediate in fish fed WCO. There were no significant differences in weight gain or specific growth rate (SGR) other than it being slightly lower in fish fed ECO compared to fish fed FO (Table 3). There were no differences in the hepatosomatic or viscerosomatic index (HSI and VSI, respectively), although values tended to be lowest in fish fed DCO and ECO (*p* = 0.576 and 0.869 respectively). No differences were observed in other fish performance parameters including feed conversion ratio (FCR) or *k* condition factor (Table 3).

**Table 3:** Growth performance, survival, feed utilization and basic and whole body proximate composition (% dry weight) of gilthead sea bream after 11 weeks of feeding the experimental diets

	FO	WCO	ECO	DCO
Initial weight (g)	55.4 ± 6.5	55.8 ± 6.5	55.0 ± 7.2	56.0 ± 6.1
Final weight (g)	129.9 ± 10.8^a^	123.2 ± 15.5^ab^	117.3 ± 13.6^b^	126.9 ± 20.0^a^
Final length (cm)	18.3 ± 0.5^a^	18.0 ± 0.7^ab^	17.8 ± 0.6^b^	18.3 ± 0.9^a^
Weight gain (g)	74.5 ± 2.3	73.5 ± 9.7	62.2 ± 2.6	71.1 ± 4.1
HSI	1.4 ± 0.3	1.6 ± 0.3	1.4 ± 0.2	1.5 ± 0.2
VSI	8.0 ± 1.4	8.0 ± 1.0	7.8 ± 0.5	7.9 ± 1.0
FI (g/tank)	3482.1 ± 215.5^a^	3108.1 ± 88.9^ab^	2898.1 ± 130.3^b^	3465.9 ± 127.9^a^
FCR	1.3 ± 0.1	1.2 ± 0.2	1.3 ± 0.1	1.4 ± 0.1
SGR	1.0 ± 0.1^a^	1.0 ± 0.2^ab^	0.9 ± 0.1^b^	1.0 ± 0.2^ab^
*k*	1.2 ± 0.1	1.1 ± 0.2	1.1 ± 0.1	1.1 ± 0.2
Whole body composition (% dry wt.)				
Crude protein	47.5 ± 0.7	48.0 ± 1.7	49.6 ± 1.3	49.3 ± 0.5
Crude lipid	35.7 ± 2.0	38.4 ± 1.6	38.5 ± 1.2	37.3 ± 0.3
Ash	9.8 ± 0.9	9.8 ± 0.6	9.8 ± 0.5	9.9 ± 0.2

Data are expressed as mean ± SD (*n* = 3). Different superscript letters within a row denote significant differences among diets as determined by one-way ANOVA with Tukey’s comparison test (*p* < 0.005)

*DCO* feed containing EPA + DHA oil from transgenic Camelina, *ECO* feed containing high-EPA oil from transgenic Camelina, *FI* feed intake, *FO* fish oil feed, *HSI* hepatosomatic index, *k* condition factor, *SGR* specific growth rate, *VSI* viscerosomatic index, *WCO* wild-type Camelina oil feed

### Lipid and Fatty Acid Digestibility

**Fig. 1:**
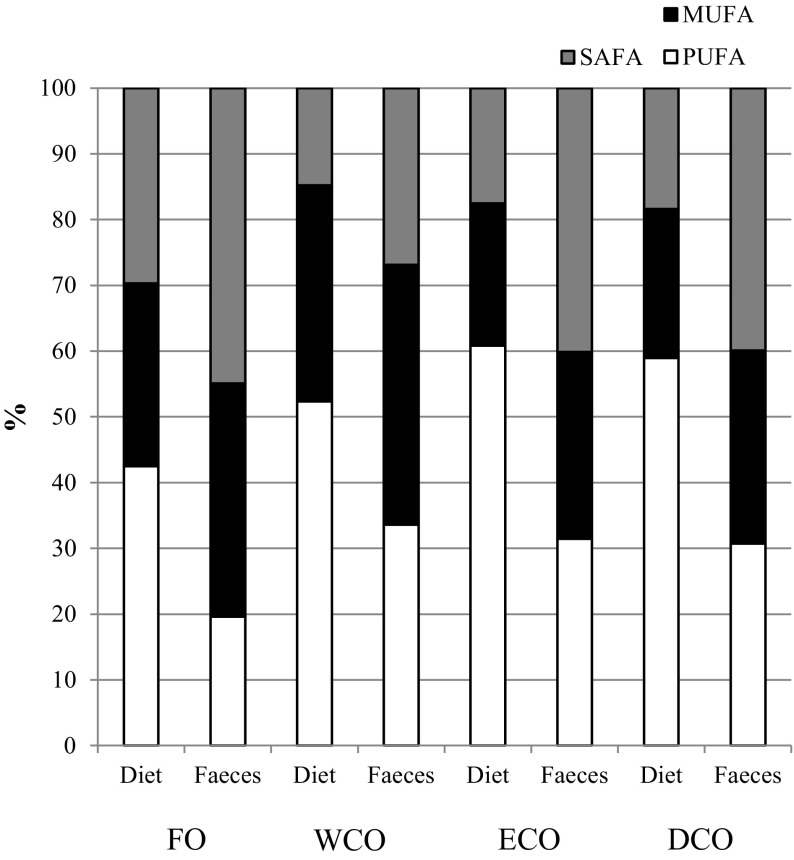
Fatty acid compositions (mol%) of the four experimental feeds and faeces showing preferential order of absorption with differing degree of unsaturation of dietary fatty acids

The apparent digestibility coefficients (ADC) were calculated for lipids and fatty acids using yttrium oxide as an inert marker. There were no significant differences in crude lipid ADC among the dietary treatments (Table 4). Figure 1 represents the analysed fatty acid composition of feeds and faeces in order to compare which fatty acid groups were preferentially digested and absorbed (*i.e.* those found in lower amounts in faeces). There was a trend for higher proportions of saturated fatty acids (SAFA) in faeces relative to the feeds, with the ADC for SAFA varying between around 81 and 87 %, generally slightly lower than the ADCs for the other fatty acids (Table 4). In contrast, proportions of PUFA in the faeces were generally lower compared to proportions in feeds with ADCs ranging from 93 to almost 97 %, whereas proportions of monounsaturated fatty acid (MUFA) were similar in diet and faeces with ADC ranging from around 83 to 92 % (Fig. 1). Some variations were found between feeds, with ADC of SAFA being highest in fish fed FO and lowest in fish fed ECO, with ADC of MUFA being higher in fish fed ECO and DCO compared to fish fed FO (Table 4). Similarly, ADC of 18:2n-6 and n-6 PUFA were higher in fish fed DCO compared to fish fed FO. Digestibility was highest with n-3 PUFA generally although ADC for EPA was lowest in WCO, although not different to that of ECO. Regarding DHA, FO and DCO showed the highest ADC, being over 96 % (Table 4).

**Table 4:** Apparent digestibility coefficient (ADC) of lipid and fatty acids in gilthead sea bream fed the four experimental diets differing in oil source

	FO	WCO	ECO	DCO
Lipid ADC	83.8 ± 4.1	81.2 ± 6.3	80.5 ± 8.0	85.8 ± 5.2
14:0	88.8 ± 3.4	80.2 ± 10.3	84.0 ± 8.5	87.1 ± 6.1
15:0	99.0 ± 0.2	97.7 ± 0.9	97.5 ± 1.1	98.2 ± 0.6
16:0	83.3 ± 4.0	78.5 ± 8.0	78.5 ± 8.8	83.4 ± 5.5
18:0	78.9 ± 3.9	75.8 ± 5.7	77.3 ± 0.2	78.5 ± 4.3
Total saturated^A^	87.4 ± 1.3^a^	86.5 ± 1.4^a^	81.3 ± 0.4^c^	84.2 ± 0.5^b^
16:1n-7	89.4 ± 4.8	83.3 ± 5.0	90.2 ± 0.2	90.8 ± 1.4
18:1n-9	81.8 ± 8.3	82.9 ± 9.1	86.9 ± 8.1	84.7 ± 8.6
18:1n-7	86.2 ± 5.8	84.7 ± 6.7	86.2 ± 7.4	88.9 ± 6.1
20:1n-9	89.6 ± 4.0	89.8 ± 3.9	88.6 ± 6.3	92.7 ± 3.1
20:1n-7	87.4 ± 3.7	86.2 ± 4.6	89.0 ± 1.4	89.5 ± 3.4
22:1n-11	93.2 ± 1.6^a^	87.2 ± 4.5^b^	88.8 ± 1.5^ab^	92.3 ± 0.1^a^
22:1n-9	78.3 ± 7.9	83.1 ± 5.0	84.0 ± 2.7	84.7 ± 4.6
Total monoenes^B^	83.4 ± 1.2^b^	87.9 ± 3.5^ab^	89.6 ± 0.5^a^	91.9 ± 1.0^a^
18:2n-6	90.6 ± 2.9^b^	92.6 ± 2.3^ab^	95.0 ± 0.1^ab^	95.8 ± 0.6^a^
20:2n-6	88.8 ± 3.1	89.5 ± 4.3	93.2 ± 0.7	92.6 ± 4.0
20:4n-6	97.4 ± 0.1	92.9 ± 1.2	96.1 ± 2.3	95.6 ± 1.5
Total n-6 PUFA^C^	92.1 ± 2.3^b^	94.0 ± 0.7^ab^	95.1 ± 0.1^ab^	96.1 ± 0.5^a^
18:3n-3	92.7 ± 2.0^b^	95.0 ± 1.8^ab^	96.4 ± 0.3^a^	97.1 ± 0.4^a^
18:4n-3	97.6 ± 0.4^a^	92.9 ± 2.0^b^	97.5 ± 0.1^a^	98.1 ± 0.3^a^
20:3n-3	96.0 ± 4.0	90.8 ± 3.5	94.4 ± 0.1	95.4 ± 0.5
20:4n-3	95.2 ± 1.1^a^	82.2 ± 5.1^b^	97.1 ± 0.1^a^	96.9 ± 0.3^a^
20:5n-3	98.2 ± 0.3^a^	95.5 ± 0.9^b^	97.3 ± 0.0^ab^	98.0 ± 0.1^a^
22:5n-3	93.4 ± 1.5^a^	80.4 ± 2.2^b^	87.8 ± 4.1^ab^	92.2 ± 5.1^a^
22:6n-3	96.8 ± 1.2^a^	93.6 ± 1.2^b^	93.6 ± 1.8^b^	96.6 ± 0.1^a^
Total n-3 PUFA	97.4 ± 0.4^a^	95.0 ± 1.9^b^	96.6 ± 0.3^ab^	97.1 ± 0.3^ab^
Total PUFA^D^	96.4 ± 0.7^a^	93.0 ± 2.3^b^	95.9 ± 0.2^ab^	96.7 ± 0.4^a^

Data expressed as mean ± SD (*n* = 3). Different superscript letters within a row denote significant differences among diets. Statistical differences were determined by one-way ANOVA with Tukey’s comparison test (*p* < 0.05)

*DCO* feed containing EPA + DHA oil from transgenic Camelina, *ECO* feed containing oil from transgenic Camelina, *FO* fish oil feed, *WCO* wild-type Camelina oil feed

^A^Includes 22:0 and 24:0

^B^Includes 16:1n-9 and 24:1n-9

^C^Includes 22:4n-6 and 22:5n-6

^D^Includes C_16_ PUFA

### Whole Fish Composition

No differences were found in any of the analysed components among fish fed the four dietary treatments (Table 3). However, there were trends for higher lipid content in fish fed the vegetable-based feeds (*p* = 0.186), and increased protein in fish fed the ECO and DCO feeds containing oil from transgenic *Camelina* (*p* = 0.202).

### Tissue Lipid Content

The lipid content of flesh (muscle) varied between around 3 and 4.5 % but diet had no significant effect (Table 5). In contrast, the lipid content of liver was higher in fish fed WCO than in fish fed FO with fish fed the oils derived from transgenic Camelina showing intermediate values (Table 6). Diet had no significant effect on the lipid content of other tissues including gill (Table 6), anterior intestine and brain (Table 7).

**Table 5:** Total lipid content (percentage of wet weight) and total lipid fatty acid composition (mol%) of muscle (flesh) of sea bream after feeding the experimental diets for 11 weeks

	FO	WCO	ECO	DCO
Lipid content (%)	4.5 ± 0.6	3.6 ± 0.6	2.9 ± 0.6	3.5 ± 0.5
Fatty acid composition				
14:0	4.6 ± 0.0^a^	3.0 ± 1.3^ab^	2.4 ± 0.1^b^	2.2 ± 0.1^b^
16:0	19.6 ± 0.3	17.4 ± 2.5	16.5 ± 0.2	16.6 ± 0.3
18:0	4.3 ± 0.2^ab^	4.1 ± 0.3^b^	4.6 ± 0.2^ab^	4.8 ± 0.3^a^
20:0	0.2 ± 0.0^b^	0.4 ± 0.2^b^	0.7 ± 0.0^a^	0.6 ± 0.0^a^
Total saturated^A^	29.3 ± 0.3	25.2 ± 3.9	24.7 ± 0.3	24.7 ± 0.4
16:1n-7	6.8 ± 0.1^a^	4.4 ± 2.1^ab^	3.3 ± 0.2^b^	3.2 ± 0.2^b^
18:1n-9	19.9 ± 0.6^a^	20.2 ± 0.7^a^	17.1 ± 0.8^b^	17.7 ± 0.4^b^
18:1n-7	3.4 ± 0.2^a^	2.5 ± 0.7^ab^	2.5 ± 0.0^ab^	2.4 ± 0.1^b^
20:1n-11	0.3 ± 0.0^a^	0.2 ± 0.1^b^	0.2 ± 0.0^b^	0.2 ± 0.0^b^
20:1n-9	1.4 ± 0.0	4.2 ± 2.4	3.2 ± 0.1	3.4 ± 0.1
20:1n-7	0.2 ± 0.0	0.2 ± 0.0	0.3 ± 0.0	0.2 ± 0.0
22:1n-11	1.2 ± 0.0^a^	1.0 ± 0.3^ab^	0.9 ± 0.0^b^	0.9 ± 0.0^b^
22:1n-9	0.4 ± 0.0	1.0 ± 0.5	0.7 ± 0.0	0.7 ± 0.0
Total monounsaturated^B^	34.9 ± 0.5^a^	35.0 ± 1.1^a^	29.2 ± 1.1^b^	30.0 ± 0.7^b^
18:2n-6	6.6 ± 0.3^b^	11.1 ± 4.1^a^	14.6 ± 0.2^a^	13.6 ± 0.3^a^
18:3n-6	0.2 ± 0.0^c^	0.2 ± 0.0^c^	0.6 ± 0.0^b^	1.8 ± 0.1^a^
20:2n-6	0.2 ± 0.0^b^	0.8 ± 0.1^ab^	1.0 ± 0.0^a^	0.6 ± 0.0^ab^
20:3n-6	0.2 ± 0.0^b^	0.3 ± 0.1^b^	0.9 ± 0.1^a^	1.0 ± 0.1^a^
20:4n-6	0.9 ± 0.0^bc^	0.6 ± 0.3^c^	1.7 ± 0.2^a^	1.3 ± 0.1^b^
22:4n-6	0.1 ± 0.0^bc^	0.1 ± 0.0^c^	0.2 ± 0.0^a^	0.1 ± 0.0^b^
Total n-6 PUFA^C^	8.5 ± 0.3^b^	13.2 ± 4.3^ab^	19.2 ± 0.2^a^	18.7 ± 0.1^a^
18:3n-3	1.2 ± 0.0^d^	12.1 ± 0.1^a^	4.6 ± 0.2^c^	5.8 ± 0.1^b^
18:4n-3	1.1 ± 0.0^b^	0.6 ± 0.0^d^	0.7 ± 0.0^c^	1.7 ± 0.1^a^
20:3n-3	0.1 ± 0.0	0.6 ± 0.5	0.5 ± 0.0	0.4 ± 0.0
20:4n-3	0.7 ± 0.0^b^	0.6 ± 0.1^b^	1.4 ± 0.0^a^	1.5 ± 0.1^a^
20:5n-3	8.0 ± 0.1^a^	4.5 ± 3.1^c^	7.8 ± 0.3^a^	5.7 ± 0.1^b^
22:5n-3	2.9 ± 0.1^a^	1.3 ± 0.1^c^	2.9 ± 0.2^a^	2.2 ± 0.1^b^
22:6n-3	10.8 ± 0.1^a^	7.4 ± 0.9^c^	8.3 ± 0.9^bc^	9.7 ± 0.6^ab^
Total n-3 PUFA	25.2 ± 0.3	25.6 ± 1.4	26.3 ± 1.2	26.1 ± 0.5
Total PUFA^D^	35.8 ± 0.6^c^	39.8 ± 4.1^bc^	46.1 ± 1.3^a^	45.3 ± 0.5^ab^
EPA + DHA	18.8 ± 0.2^a^	12.0 ± 3.2^b^	16.1 ± 1.2^ab^	14.4 ± 0.5^ab^
EPA/DHA	0.7 ± 0.0	0.6 ± 0.4	0.9 ± 0.1	0.5 ± 0.0
EPA + DPA + DHA	21.7 ± 0.2^a^	13.3 ± 3.3^b^	19.0 ± 1.3^a^	16.6 ± 0.6^ab^

Data are expressed as mean ± SD (*n* = 3). Different superscript letters within a row denote significant differences among diets as determined by one-way ANOVA with Tukey’s comparison test (*p* < 0.005)

*DCO* feed containing EPA + DHA oil from transgenic Camelina, *DHA* docosahexaenoic acid (22:6n-3); DPA, docosapentaenoic acid (22:5n-3), *ECO* feed containing high-EPA oil from transgenic Camelina, *EPA* eicosapentaenoic acid (20:5n-3), *FO* fish oil feed, *WCO* wild-type Camelina oil feed

^A^Includes 15:0, 22:0 and 24:0

^B^Includes 16:1n-9 and 24:1n-9

^C^Includes 22:5n-6

^D^Includes C_16_ PUFA

**Table 6:** Total lipid content (percentage of wet weight) and fatty acid compositions (mol%) of total lipid of liver and gills of sea bream

	FO	WCO	ECO	DCO
Liver				
Lipid content	7.6 ± 1.6^b^	10.4 ± 1.6^a^	8.8 ± 0.1^ab^	8.6 ± 0.6^ab^
Fatty acid composition				
16:0	20.8 ± 0.4^a^	18.1 ± 1.3^b^	16.7 ± 0.9^b^	18.1 ± 1.3^b^
Total saturated^A^	31.7 ± 0.7^a^	26.5 ± 1.8^b^	25.9 ± 1.5^b^	27.7 ± 1.8^b^
18:1n-9	21.2 ± 1.0^ab^	23.6 ± 1.8^a^	17.9 ± 1.4^b^	18.6 ± 1.8^b^
Total monounsaturated^B^	35.1 ± 1.7^ab^	37.3 ± 2.4^a^	29.2 ± 1.9^b^	30.2 ± 2.4^b^
18:2n-6	4.6 ± 0.7^c^	10.9 ± 0.7^b^	13.6 ± 0.6^a^	12.0 ± 0.7^ab^
20:4n-6	1.3 ± 0.2^b^	0.6 ± 0.2^c^	2.2 ± 0.3^a^	1.7 ± 0.2^ab^
22:4n-6	0.1 ± 0.1^bc^	0.0 ± 0.0^c^	0.2 ± 0.0^a^	0.2 ± 0.0^a^
Total n-6 PUFA^C^	7.0 ± 0.6^c^	13.8 ± 0.9^b^	19.4 ± 1.1^a^	17.9 ± 0.9^a^
18:3n-3	0.8 ± 0.1^c^	9.8 ± 0.5^a^	4.2 ± 0.3^b^	5.2 ± 0.5^b^
20:5n-3	7.4 ± 0.4^a^	2.1 ± 0.6^c^	6.9 ± 0.7^a^	3.9 ± 0.6^b^
22:5n-3	2.9 ± 0.1^a^	0.9 ± 0.2^c^	3.2 ± 0.4^a^	1.9 ± 0.2^b^
22:6n-3	11.8 ± 1.4^a^	6.5 ± 1.7^b^	7.9 ± 0.7^b^	9.2 ± 1.7^ab^
Total n-3 PUFA	24.7 ± 1.7	22.2 ± 3.3	25.1 ± 2.4	23.9 ± 3.3
Total PUFA^D^	33.2 ± 1.6^a^	36.2 ± 4.2^ab^	44.9 ± 3.4^a^	42.2 ± 4.2^ab^
EPA + DHA	19.1 ± 1.8^a^	8.6 ± 2.3^c^	14.7 ± 1.4^ab^	13.2 ± 2.3^bc^
EPA/DHA	0.6 ± 0.0^b^	0.3 ± 0.0^d^	0.9 ± 0.0^a^	0.4 ± 0.0^c^
EPA + DPA + DHA	22.1 ± 1.9^a^	9.5 ± 2.5^c^	17.9 ± 1.8^ab^	15.1 ± 2.5^b^
Gills				
Lipid content	10.5 ± 1.5	9.9 ± 1.7	12.1 ± 1.0	11.7 ± 1.5
Fatty acid composition				
16:0	19.0 ± 0.1^a^	15.6 ± 0.6^b^	15.6 ± 0.5^b^	15.8 ± 0.2^b^
Total saturated^A^	28.3 ± 0.2^a^	22.6 ± 0.7^b^	23.3 ± 0.6^b^	23.4 ± 0.5^b^
18:1n-9	20.4 ± 0.3^ab^	21.8 ± 0.9^a^	18.7 ± 0.7^b^	19.7 ± 0.6^b^
Total monounsaturated^B^	36.0 ± 0.2^a^	37.1 ± 1.1^a^	31.9 ± 0.9^b^	32.9 ± 0.8^b^
18:2n-6	7.2 ± 0.1^c^	13.4 ± 0.4^b^	15.1 ± 0.2^a^	13.8 ± 0.4^b^
20:4n-6	0.9 ± 0.1^b^	0.4 ± 0.0^c^	1.5 ± 0.1^a^	1.1 ± 0.0^ab^
22:4n-6	0.1 ± 0.0^b^	0.0 ± 0.0^c^	0.2 ± 0.0^a^	0.2 ± 0.0^b^
Total n-6 PUFA^C^	9.2 ± 0.2^c^	15.4 ± 0.5^b^	19.4 ± 0.4^a^	18.4 ± 0.6^a^
18:3n-3	1.3 ± 0.0^c^	11.0 ± 0.6^a^	4.6 ± 0.3^b^	5.6 ± 0.3^b^
20:5n-3	7.5 ± 0.1^a^	2.8 ± 0.1^c^	7.0 ± 0.3^a^	4.5 ± 0.1^b^
22:5n-3	2.8 ± 0.1^a^	1.3 ± 0.1^c^	2.7 ± 0.1^a^	2.2 ± 0.0^b^
22:6n-3	10.6 ± 0.2^a^	6.9 ± 0.5^c^	7.4 ± 0.2^c^	8.8 ± 0.3^b^
Total n-3 PUFA	24.4 ± 0.3	24.2 ± 1.2	24.7 ± 1.1	24.7 ± 0.2
Total PUFA^D^	35.6 ± 0.2^c^	40.3 ± 1.7^b^	44.8 ± 1.4^a^	43.7 ± 0.8^a^
EPA + DHA	18.0 ± 0.2^a^	9.7 ± 0.6^d^	14.5 ± 0.4^b^	13.3 ± 0.3^c^
EPA/DHA	0.7 ± 0.0^b^	0.4 ± 0.0^d^	0.9 ± 0.0^a^	0.5 ± 0.0^c^
EPA + DPA + DHA	20.8 ± 0.2^a^	11.0 ± 0.6^d^	17.2 ± 0.6^b^	15.5 ± 0.3^c^

Data are expressed as mean ± SD (*n* = 3). Different superscript letters within a row denote significant differences among diets as determined by one-way ANOVA with Tukey’s comparison test (*p* < 0.005)

*DCO* feed containing EPA + DHA oil from transgenic Camelina, *DHA* docosahexaenoic acid (22:6n-3), *DPA* docosapentaenoic acid (22:5n-3), *ECO* feed containing high-EPA oil from transgenic Camelina, *EPA* eicosapentaenoic acid (20:5n-3), *FO* fish oil feed, *WCO* wild-type Camelina oil feed

^A^Includes 15:0, 22:0 and 24:0

^B^Includes 16:1n-9 and 24:1n-9

^C^Includes 22:5n-6

^D^Includes C_16_ PUFA

**Table 7:** Total lipid content (percentage of wet weight) and fatty acid compositions (mol%) of total lipid of anterior intestine and brain of sea bream

	FO	WCO	ECO	DCO
Anterior intestine				
Lipid content	6.9 ± 1.8	5.0 ± 1.4	4.5 ± 1.0	5.0 ± 0.2
Fatty acid composition				
16:0	19.7 ± 0.1^a^	13.4 ± 0.8^b^	14.6 ± 0.5^b^	15.0 ± 0.8^b^
Total saturated^A^	33.1 ± 1.3^a^	22.1 ± 1.9^c^	25.3 ± 0.2^bc^	27.1 ± 1.5^b^
18:1n-9	15.3 ± 1.5^ab^	17.8 ± 1.3^a^	14.5 ± 0.5^ab^	8.3 ± 7.0^b^
Total monounsaturated^B^	30.6 ± 2.5^a^	32.9 ± 1.7^a^	26.7 ± 0.6^b^	31.7 ± 3.8^ab^
18:2n-6	5.7 ± 0.8^b^	15.4 ± 0.6^a^	16.2 ± 0.7^a^	16.6 ± 2.1^a^
20:2n-6	0.2 ± 0.0^c^	1.3 ± 0.1^a^	1.2 ± 0.1^a^	0.8 ± 0.1^b^
20:3n-6	0.2 ± 0.0^c^	0.2 ± 0.0^c^	1.0 ± 0.0^b^	1.3 ± 0.2^a^
20:4n-6	1.7 ± 0.4^b^	0.8 ± 0.2^b^	3.0 ± 0.2^a^	2.9 ± 0.7^a^
22:4n-6	0.1 ± 0.0^b^	0.0 ± 0.0^c^	0.3 ± 0.0^a^	0.2 ± 0.0^a^
Total n-6 PUFA^C^	8.4 ± 0.4^c^	18.0 ± 0.5^b^	22.3 ± 1.0^a^	24.0 ± 2.3^a^
18:3n-3	0.9 ± 0.2^c^	13.9 ± 1.7^a^	5.1 ± 0.5^b^	6.9 ± 1.3^b^
20:5n-3	8.1 ± 0.5^a^	2.6 ± 0.3^d^	7.0 ± 0.2^b^	4.2 ± 0.2^c^
22:5n-3	2.3 ± 0.2^a^	1.0 ± 0.1^c^	2.6 ± 0.3^a^	1.7 ± 0.1^b^
22:6n-3	12.4 ± 2.3^a^	7.4 ± 1.5^b^	7.8 ± 0.4^b^	10.8 ± 1.3^ab^
Total n-3 PUFA	25.7 ± 2.1	26.6 ± 0.1	25.1 ± 0.5	26.8 ± 0.9
Total PUFA^D^	36.3 ± 1.6^c^	45.0 ± 0.5^b^	47.9 ± 0.4^ab^	51.2 ± 3.1^a^
EPA + DHA	20.6 ± 2.6^a^	9.9 ± 1.8^c^	14.9 ± 0.6^b^	15.0 ± 1.1^b^
EPA/DHA	0.7 ± 0.1^b^	0.4 ± 0.0^c^	0.9 ± 0.0^a^	0.4 ± 0.1^c^
EPA + DPA + DHA	22.9 ± 2.5^a^	10.9 ± 1.8^c^	17.5 ± 0.9^b^	16.8 ± 1.0^b^
Brain				
Lipid content	9.6 ± 0.2	9.6 ± 0.7	8.9 ± 1.0	8.1 ± 0.2
Fatty acid composition				
16:0	19.1 ± 0.6	19.3 ± 0.1	18.9 ± 0.8	18.6 ± 1.3
Total saturated^A^	34.3 ± 0.9	34.3 ± 0.3	34.4 ± 0.4	33.9 ± 1.0
18:1n-9	21.4 ± 0.5^a^	21.3 ± 0.5^a^	20.4 ± 0.6^ab^	20.2 ± 0.5^b^
Total monounsaturated^B^	34.4 ± 1.1	33.8 ± 0.6	32.7 ± 1.4	32.6 ± 0.7
18:2n-6	2.0 ± 0.8	2.8 ± 0.5	3.1 ± 0.6	3.5 ± 0.6
20:2n-6	0.1 ± 0.1^b^	0.2 ± 0.0^a^	0.2 ± 0.1^a^	0.2 ± 0.0^a^
20:3n-6	0.1 ± 0.0^d^	0.2 ± 0.0^c^	0.3 ± 0.0^b^	0.4 ± 0.0^a^
20:4n-6	1.7 ± 0.1^b^	1.5 ± 0.1^b^	2.0 ± 0.1^a^	2.0 ± 0.1^a^
22:4n-6	0.2 ± 0.0^ab^	0.1 ± 0.0^b^	0.2 ± 0.0^a^	0.2 ± 0.0^ab^
Total n-6 PUFA^C^	4.1 ± 0.8^b^	4.8 ± 0.5^ab^	5.9 ± 0.7^a^	6.5 ± 0.7^a^
18:3n-3	0.4 ± 0.1^b^	1.7 ± 0.5^a^	0.9 ± 0.3^ab^	1.3 ± 0.3^ab^
20:5n-3	4.6 ± 0.3^a^	3.6 ± 0.2^b^	4.3 ± 0.1^a^	3.7 ± 0.1^b^
22:5n-3	1.6 ± 0.1^a^	1.2 ± 0.0^b^	1.5 ± 0.1^a^	1.3 ± 0.0^b^
22:6n-3	20.0 ± 0.9	20.0 ± 0.5	19.8 ± 0.7	20.1 ± 0.8
Total n-3 PUFA	27.0 ± 0.5	27.0 ± 0.5	27.0 ± 1.0	27.0 ± 0.3
Total PUFA^D^	31.3 ± 0.9	31.9 ± 0.8	32.9 ± 1.6	33.5 ± 0.6
EPA + DHA	24.6 ± 0.7	23.7 ± 0.8	24.0 ± 0.8	23.8 ± 0.7
EPA/DHA	0.2 ± 0.0	0.2 ± 0.0	0.2 ± 0.0	0.2 ± 0.0
EPA + DPA + DHA	26.2 ± 0.6	24.9 ± 0.8	25.6 ± 0.7	25.1 ± 0.6

Data are expressed as mean ± SD (*n* = 3). Different superscript letters within a row denote significant differences among diets as determined by one-way ANOVA with Tukey’s comparison test (*p* < 0.005)

*DCO* feed containing EPA + DHA oil from transgenic Camelina, *DHA* docosahexaenoic acid (22:6n-3), *DPA* docosapentaenoic acid (22:5n-3), *ECO* feed containing high-EPA oil from transgenic Camelina, *EPA* eicosapentaenoic acid (20:5n-3), *FO* fish oil feed, *WCO* wild-type Camelina oil feed

^A^Includes 15:0, 22:0 and 24:0

^B^Includes 16:1n-9 and 24:1n-9

^C^Includes 22:5n-6

^D^Includes C_16_ PUFA

### Tissue Fatty Acid Compositions

#### Muscle (Flesh)

No differences were observed in the proportions of total n-3 PUFA in muscle (*p* = 0.508), although clear differences were found in individual fatty acids (Table 5). The mole percentages of EPA were highest in fish fed FO and ECO, lowest in fish fed WCO and intermediate in fish fed DCO. Proportions of DHA were similar in fish fed FO and DCO and higher than in fish fed WCO, with fish fed ECO showing intermediate values. Fish fed the FO and ECO diets displayed similar proportions of n-3 docosapentaenoic acid (DPA, 22:5n-3) in flesh, which were significantly higher than those found in fish fed WCO or DCO. The totals of EPA + DHA and EPA + DPA + DHA in fish fed all feeds varied in the rank order FO > ECO > DCO > WCO. The proportions of 18:2n-6 and total n-6 PUFA were higher in flesh of sea bream fed all the diets containing VO (WCO, ECO and DCO) compared to fish fed FO (Table 5). No differences were observed in total SAFA and total MUFA were higher in fish fed FO and WCO compared to fish fed the oils from transgenic *Camelina*.

#### Liver

The fatty acid profile of liver was similar to that of muscle and mainly reflected dietary compositions. No differences were found in total n-3 PUFA among the four dietary treatments as low levels of n-3 LC-PUFA were associated with high levels of short chain precursors (Table 6). The percentages of EPA were similar and highest in fish fed FO and ECO, lowest in fish fed WCO and intermediate in fish fed DCO. Proportions of DHA were similar in fish fed FO and DCO and higher than in fish fed WCO, with fish fed ECO showing intermediate values. Again, the totals of EPA + DHA and EPA + DPA + DHA in fish fed all feeds were in the rank order FO > ECO > DCO > WCO. Proportions of total n-6 PUFA were higher in liver of fish-fed ECO and DCO reflecting the higher dietary n-6 contents, particularly 18:2n-6 and arachidonic acid (ARA, 20:4n-6).

#### Gills

In general terms, gill fatty acid compositions mirrored dietary input, although some small differences were found (Table 6). Proportions of EPA varied in the rank order FO = ECO > DCO > WCO, DHA in the rank order FO > DCO > ECO = WCO, and EPA + DHA and EPA + DPA + DHA in the rank order FO > ECO > DCO > WCO. 18:2n-6 was higher in gills of fish fed the VO diets compared to fish fed FO while ARA varied in the rank order ECO > DCO = FO > WCO (Table 6).

#### Anterior Intestine

Proportions of EPA, DHA and total EPA + DPA + DHA varied in anterior intestine with essentially the same pattern as described for gills (Table 7). However, total n-6 PUFA contents were higher in anterior intestine than in the other tissues analysed, particularly in fish fed the diets containing oil from transgenic Camelina due to higher levels of 18:2n-6 and ARA.

#### Brain

The fatty acid composition of brain was least influenced by diet, with fewer individual fatty acids showing significant differences and the magnitude of differences being lower (Table 7). Specifically and importantly, DHA, EPA + DHA, EPA + DPA + DHA and EPA/DHA ratio did not vary between fish fed the different diets, and neither did 18:2n-6 or the totals of n-3 PUFA, PUFA, SAFA and MUFA.

**Fig. 2:**
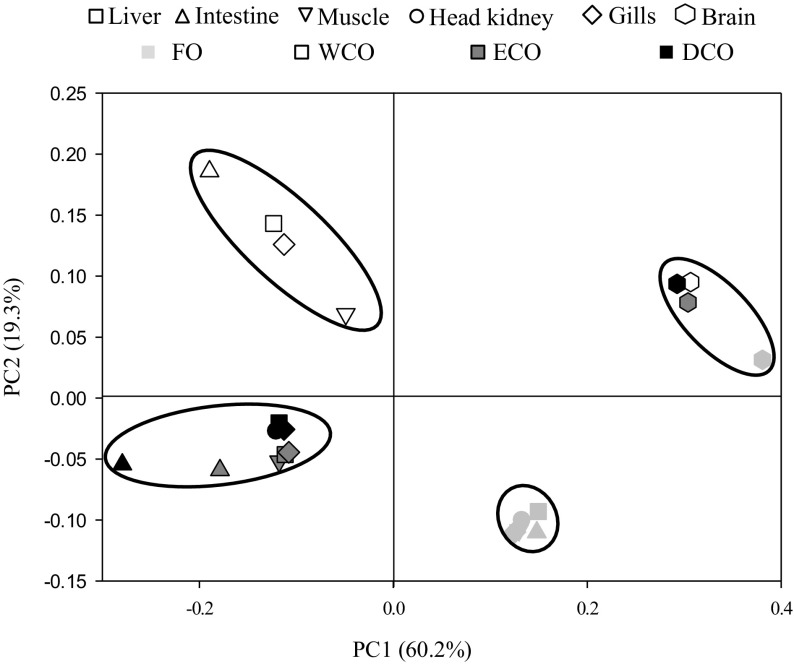
Non-metric multidimensional scaling (NMDS) plot based on logarithmically transformed fatty acid composition of liver, muscle, gill, anterior intestine and brain from gilthead sea bream fed the four dietary treatments for 11 weeks

The non-metric multidimensional scaling (NMDS) plot clearly showed that tissue fatty acid compositions were affected by the dietary input rather than by tissue type with the exception of brain, which clustered in a different group (Fig. 2, stress 0.07). This was confirmed by the global R value and low p obtained (*R* = 0.481; *p* < 0.001) when comparing the feeds. Pair-wise *R* indicated that the segregation was strongest between fish fed FO and WCO (*R* = 0.711; *p* = 0.005), whereas the weakest separation was observed between fish fed the ECO and DCO diets (*R* = 0.233; *p* = 0.030). The tissue fatty acid profiles of fish fed the two transgenic-derived oils did not show high separation with WCO (*R* = 0.431 and 0.404, and *p* = 0.003 and 0.004 for ECO and DCO, respectively). Tissue-wise, a weak segregation was observed (global *R* = 0.261; *p* = 0.008), although brain displayed a strong separation from other tissues (*i.e. R* = 1 for muscle and 0.958 for gill).

### Tissue Histology

Fish fed the FO diet showed regular hepatocyte morphology with large centrally located nuclei with few cytoplasmic vacuoles that did not alter hepatocyte shape or size (Table 8). Fish fed wild-type Camelina oil (WCO) displayed a higher degree of vacuolisation, although no structural changes, such as inflammation, necrosis or perivascular cuffing were observed. Fish fed the feeds containing the GM-derived oils showed intermediate levels of vacuolisation, with no differences between FO and DCO-fed fish or between WCO and ECO-fed fish (Table 8). No significant differences were observed in the infiltration of peripancreatic fat although fish fed FO showed the lowest scores and DCO-fed fish the highest (*p* = 0.293; Table 8). With intestinal tissue, good integrity of the absorptive membrane was observed in the sections from fish fed all the dietary treatments. However, cellular infiltration, mainly represented by acidophilic granulocytes and some lymphocytes was observed, mainly in fish fed ECO (data not shown). Most of the acidophilic granulocytes were located in the lamina propria although a few could be found in submucosa and were present both in mid and hindgut.

**Table 8:** Mean scores for the lipid vacuolisation and peripancreatic fat infiltration in liver of gilthead sea bream fed the experimental diets for 11 weeks

	FO	WCO	ECO	DCO
Cytoplasmic lipid vacuolisation	0.3 ± 0.3^c^	2.3 ± 0.7^a^	1.4 ± 0.7^ab^	0.8 ± 0.4^bc^
Peripancreatic fat	0.5 ± 0.4	1.0 ± 0.5	1.0 ± 1.2	1.4 ± 0.7

Data are expressed as mean ± SD (*n* = 6). Different superscript letters within a row denote significant differences among diets as determined by one-way ANOVA with Tukey’s comparison test (*p* < 0.005). Scoring was on a scale from 0 to 3 as described in detail in the “Materials and Methods” section with 0 = not observed, 1 = few, 2 = medium, and 3 = severe

*DCO* feed containing EPA + DHA oil from transgenic Camelina, *ECO* feed containing high-EPA oil from transgenic Camelina, *FO* fish oil feed, *WCO* wild-type Camelina oil feed

### Liver and Anterior Intestine Gene Expression

#### LC-PUFA Biosynthetic Genes

**Fig. 3:**
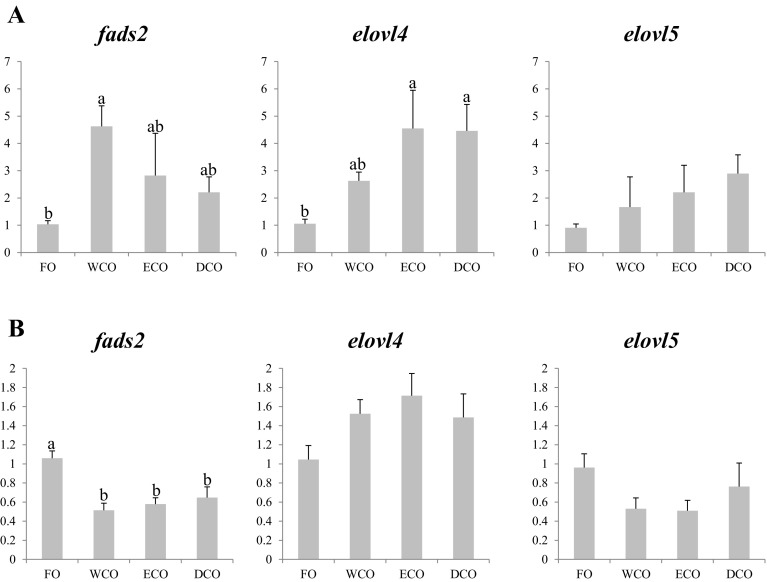
Expression, measured by qPCR of LC-PUFA biosynthesis pathway genes in sea bream liver (**a**) and anterior intestine (**b**) after eleven weeks of feeding. *Different superscript letters* denote differences in gene expression among the treatments according to one-way ANOVA (*p* < 0.05). Results are normalized expression ratios (average ± SEM; *n* = 6) of the expression of these genes in fish fed the different diets in relation to fish fed FO feed. Diets contain either fish oil (FO), wild-type Camelina oil (WCO), high-EPA Camelina oil (ECO) or EPA + DHA Camelina oil (DCO). *fads2*, delta-6-fatty acyl desaturase; *elovl4*, fatty acid elongase 4; *elovl5*, fatty acyl elongase 5

In liver, higher expression of fatty acyl desaturase 2 (*fads2*) was observed in fish fed all three diets containing VO (WCO, ECO and DCO), with expression significantly greater in liver of WCO-fed fish compared to FO-fed fish (Fig. 3a). Similarly higher expression in liver of fish fed all VO was observed in fatty acid elongase 4 (*elovl4)*, significantly so in fish fed both GM-derived oils compared to fish fed FO (Fig. 3a). There was also a non-significant trend for increased expression of fatty acid elongase 5 (*elovl5*) in fish fed VO diets compared to fish fed FO (Fig. 3a). In contrast, anterior intestine showed different nutritional regulation of these genes. Firstly, only *elovl4* expression showed a similar pattern of expression to that observed in liver with higher expression, albeit not significant, in fish fed the VO compared to fish fed FO (Fig. 3b). Secondly, fold changes (FC) were less with the highest being 1.7 FC for *elovl4* for ECO-fed fish (Fig. 3b) compared to 4.6 FC in liver for this gene in fish fed ECO (Fig. 3a). Only *fads2* showed significant regulation by dietary oil source, being down-regulated in intestine in VO-fed fish compared to fish fed FO, and a similar, non significant, trend was found in *elovl5* in intestine (Fig. 3b).

#### Lipid Metabolism Genes

**Fig. 4:**
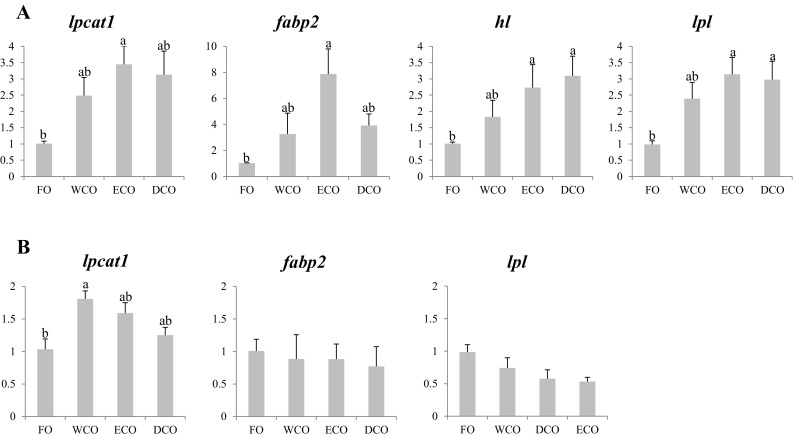
Expression, measured by qPCR of lipid metabolism genes in sea bream liver (**a**) and anterior intestine (**b**) after 11 weeks of feeding. *Different superscript letters* denote differences in gene expression among the treatments according to one-way ANOVA (*p* < 0.05). Results are normalized expression ratios (average ± SEM; *n* = 6) of the expression of these genes in fish fed the different diets in relation to fish fed FO feed. Diets contain either fish oil (FO), wild-type Camelina oil (WCO), high-EPA Camelina oil (ECO) or EPA + DHA Camelina oil (DCO). *lpcat1*, lysophosphatidylcholine acyltransferase 1; *FABP2*, fatty acid binding protein 2; *hl*, hepatic lipase; *lpl*, lipoprotein lipase

As above, the nutritional regulation of this group of genes was more marked in liver (Fig. 4a) than in anterior intestine (Fig. 4b). Lysophosphatidylcholine acyltransferase (*lpcat*) was up-regulated in VO-fed fish in both tissues, although the highest expression was found in liver of ECO-fed fish, whereas fish fed WCO showed the highest significant FC in intestine. Fatty acid binding protein 2 (*FABP2*) gene expression was also regulated in liver, with highest expression in fish fed ECO, whereas no dietary regulation of this gene was observed in anterior intestine. Both lipoprotein lipase (*lpl*) and hepatic lipase (*hl*) genes showed the same pattern in liver, with highest levels of expression in fish fed both diets with GM-derived oils, with WCO showing intermediate levels between those of ECO/DCO and fish fed FO (Fig. 4a). In contrast, no regulation was observed in the anterior intestine for *lpl*, although its expression showed a downward trend in VO-fed fish, and *hl* could not be detected in intestinal tissue (Fig. 4b).

#### Fatty Acid Catabolism Genes

**Fig. 5:**
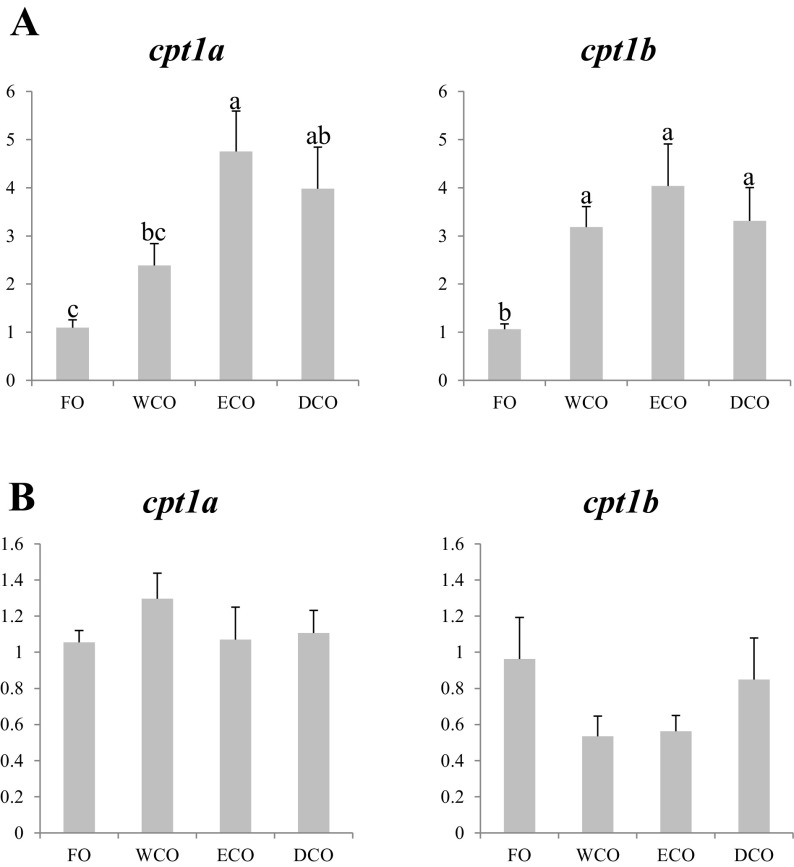
Expression, measured by qPCR of energy metabolism genes in sea bream liver (**a**) and anterior intestine (**b**) after 11 weeks of feeding. *Different superscript letters* denote differences in gene expression among the treatments according to one-way ANOVA (*p* < 0.05). Results are normalized expression ratios (average ± SEM; *n* = 6) of the expression of these genes in fish fed the different diets in relation to fish fed FO feed. Diets contain either fish oil (FO), wild-type Camelina oil (WCO), high-EPA Camelina oil (ECO) or EPA + DHA Camelina oil (DCO). *cpt1a*, carnitine palmitoyltransferase, isoform 1a; *cpt1b*, carnitine palmitoyltransferase, isoform 1b

Gene expression of two isoforms of carnitine palmitoyltransferase 1, *cpt1a* and *cpt1b*, were evaluated in liver and anterior intestine of sea bream (Fig. 5). In general terms, expression of both *cpt1* in liver was higher in fish fed the VO compared to fish fed FO. However, WCO-fed fish showed intermediate values of *cpt1a* expression, lower FC than fish fed ECO, but in the same range as DCO-fed fish (Fig. 5a). Fish fed all three VO diets showed similar and higher levels of expression of *cpt1b* in liver than fish fed FO. No significant differences in expression of either *cpt1a* or *cpt1b* were observed in anterior intestine of sea bream fed the dietary treatments (Fig. 5b).

#### Nuclear Receptors

**Fig. 6:**
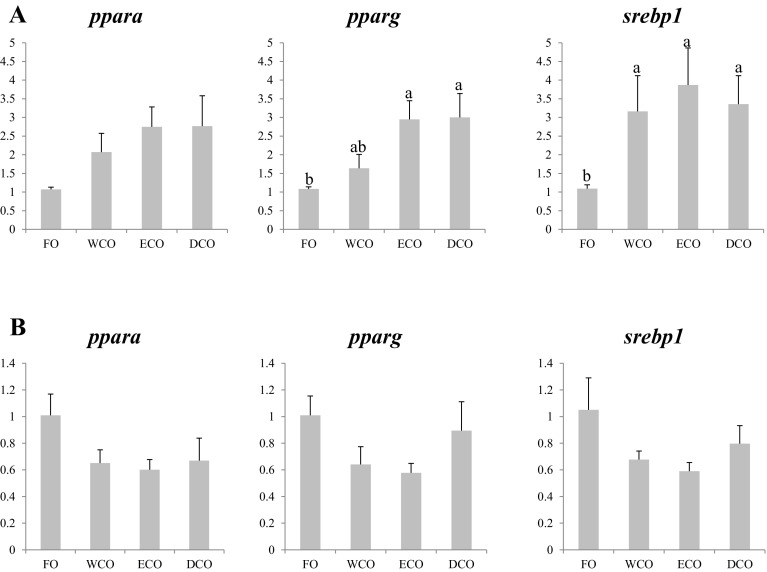
Expression, measured by qPCR of transcription factor genes in sea bream liver (**a**) and anterior intestine (**b**) after 11 weeks of feeding. *Different superscript letters* denote differences in gene expression among the treatments according to one-way ANOVA (*p* < 0.05). Results are normalized expression ratios (average ± SEM; *n* = 6) of the expression of these genes in fish fed the different diets in relation to fish fed FO feed. Diets contain either fish oil (FO), wild-type Camelina oil (WCO), high-EPA Camelina oil (ECO) or EPA + DHA Camelina oil (DCO). *PPARα*, peroxisome proliferator-activated receptor alpha; *PPARγ*, peroxisome proliferator-activated receptor gamma; *srebp1*, sterol regulatory element-binding protein 1

Liver expression of the three evaluated nuclear receptors was generally higher in fish fed all of the VO diets compared to fish fed FO, although differences were not significant for peroxisome proliferator-activated receptor α (*PPARα*) (Fig. 6a). Expression in liver of *PPARγ* was highest in ECO- and DCO-fed fish with WCO-fed fish showing intermediate values, whereas fish fed all the VO diets showed higher expression of sterol regulatory element binding protein 1 (*srebp1*) (Fig. 6a). Although there were no significant differences in the expression of these genes among the dietary treatments in anterior intestine, a consistent pattern of lower expression in fish fed the VO diets was observed for all three nuclear receptors (Fig. 6b).

#### Fish Health and Immune System Genes

**Fig. 7:**
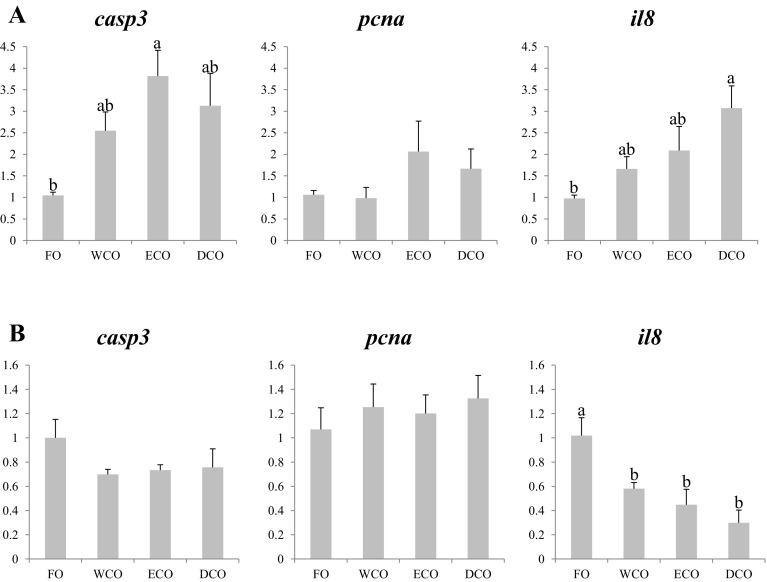
Expression, measured by qPCR of fish health and immune system genes in sea bream liver (**a**) and anterior intestine (**b**) after 11 weeks of feeding. *Different superscript letters* denote differences in gene expression among the treatments according to one-way ANOVA (*p* < 0.05). Results are normalized expression ratios (average ± SEM; *n* = 6) of the expression of these genes in fish fed the different diets in relation to fish fed FO feed. Diets contain either fish oil (FO), wild-type Camelina oil (WCO), high-EPA Camelina oil (ECO) or EPA + DHA Camelina oil (DCO). *casp3*, caspase 3; *pcna*, proliferating cell nuclear antigen; *il8*, interleukin 8

Again the three evaluated genes all showed higher expression in liver of fish fed the VO diets compared to fish fed FO (Fig. 7a). In the case of caspase 3 (*casp3*), ECO-fed fish showed the highest expression levels, although no differences were found with WCO and DCO-fed fish which also showed similar levels of expression to FO-fed fish (Fig. 7a). No statistical differences were observed for proliferating cell nuclear antigen (*pcna*) expression in liver, although DCO, and particularly ECO-fed fish, tended to have higher levels of expression. DCO-fed fish showed a clear up-regulation in hepatic interleukin 8 (*il8*) expression, with WCO and ECO-fed fish displaying intermediate levels (Fig. 7a). In contrast, although *il8* was also differentially regulated by dietary oil source in intestine, the pattern was opposite to that observed in liver, with VO-fed fish showing lower expression compared to FO-fed fish (Fig. 7b).

#### Transgenes

All analysed sea bream tissues (muscle, liver and anterior intestine) tested negative for the presence of the Camelina T-DNA gene construct as monitored by the use of npt-II primers (data not shown).

## Discussion

The replacement of FO in aquafeeds depends on finding alternative, sustainable sources of EPA and DHA, with this currently being one of the main issues in aquaculture nutrition, particularly in marine species that have limited ability for endogenous synthesis of LC-PUFA. Previous trials completely replacing FO by VO in feeds for gilthead sea bream resulted in reduced growth probably related to a reduced intake of essential LC-PUFA, which are not found in VO [31–33]. In the present study we evaluated the complete substitution of FO by two different oils obtained from GM-oilseed crops rich in either EPA (ECO) or containing both EPA and DHA (DCO) in feeds for sea bream juveniles. Both oils proved to be effective substitutes of FO, displaying growth rates that were similar to those achieved by fish fed FO (in the case of DCO) or WCO (for ECO). Similarly, recent studies employing both oil iterations as substitutes for FO in Atlantic salmon feeds showed that fish fed these oils were as successful as those fed FO [13, 15]. The lack of effect on growth in WCO-fed fish in the present trial is explained by the inclusion of relatively high levels of FM which ensured that n-3 LC-PUFA requirements were fully satisfied (1.9 % n-3 LC-PUFA in WCO-feed), estimated to be 0.9 % of dry feed [34].

It was surprising that the ECO-fed fish showed slightly reduced performance, albeit no different to WCO-fed fish, given that n-3 LC-PUFA requirements, including DHA, were satisfied. One reason for this could be related to the balance between the different dietary LC-PUFA including the dietary EPA/DHA ratio, which differed among the feeds. An EPA/DHA ratio of 2:1 was reported to be optimal for sea bream juveniles [35] and in the present trial ECO feed presented a ratio of approximately 3:1, perhaps suggesting an imbalance in these essential fatty acids. However, in previous trials in Atlantic salmon, where the EPA/DHA ratio was even higher (around 9:1), given the higher oil inclusion and lower FM level, no adverse effect was observed on growth [13]. However, it should be noted that the optimal dietary EPA/DHA ratio is likely to be species-specific. Another fatty acid that differed between ECO and the other feeds was ARA, with the ECO diet having more than double the ARA content of the FO diet. Increased dietary ARA levels have been associated with enhanced stress resistance, survival and improved growth in sea bream juveniles and larvae [36–40] as occurs in other marine warm water species such as European sea bass (*Dicentrarchus labrax*) [41]. However, ARA produces pro-inflammatory effects due to production of prostaglandin E_2_ (PGE_2_), leukotriene B_4_ (LTB_4_) and lipoxins [42], and so diets rich in n-6 PUFA, primarily ARA, could lead to overproduction of PGE_2_ although that can, in turn, have an immunosuppressant effect [43]. Although no major or obvious alteration in fish health was shown by the histology and qPCR results in the present study, increased ARA levels or even imbalanced proportions of n-3 and n-6 PUFA may partly explain the slightly lower growth of ECO-fed sea bream. On the other hand, the ratios of ARA, EPA and DHA to each other are also known to be of importance for marine finfish nutrition. For instance, a diet with high EPA and low ARA (9:1) significantly reduced performance of Atlantic salmon when compared to fish fed a more balanced ratio of EPA and ARA (1.5:1) [44], similar to what was observed in the present trial. Additionally, multiple regression analysis demonstrated meaningful relationships between ARA and DHA in California yellowtail (*Seriola dorsalis*) with ARA and DHA contributing positively to weight gain whereas EPA contributed negatively [45]. Thus, the lower growth observed in ECO-fed sea bream could be due to imbalanced proportions between these three essential LC-PUFA.

Despite the slightly reduced performance of ECO-fed fish compared to FO and DCO-fed fish, no marked differences were observed in lipid or individual fatty acid digestibility, which is consistent with previous studies in salmon using the same oil [14]. However, ECO-fed fish consumed less feed (g/tank) than fish fed FO or WCO diets, which may suggest a palatability issue with this oil. However, it must be noted that both oils were extracted using the same process and stabilized using the same concentration of antioxidant (ethoxyquin). In addition, exactly the same batch of ECO was used in the earlier trial in salmon where no differences in performance were observed between treatments [13]. Moreover, the sea bream feeds were formulated with higher FM levels than the earlier salmon feeds, which would in turn be expected to increase palatability. Thus, it appears that the slightly reduced performance of ECO-fed fish is more likely to be related to a species-specific sensitivity to high dietary ARA/n-6 levels that affected feed intake rather than a problem with palatability.

Complete substitution of dietary FO by VO is also associated with increased deposition of C_18_ fatty acids and reduced proportions of LC-PUFA in fish tissues. The two-GM derived oils investigated in the present trial can be considered as “hybrid” oils given that they contain features of both vegetable and marine oils, and the tissue fatty acid profiles reflected this characteristic. Thus, in general terms tissues of ECO and DCO fed fish had higher proportions of n-3 LC-PUFA than fish fed WCO, which in the case of flesh enhanced the nutritional value of the product. Some limited biosynthetic activity was observed particularly in fish fed ECO with high EPA (and ARA), where higher levels of DPA (22:5n-3) and 22:4n-6 were observed in liver, muscle and gills compared to fish fed either WCO or DCO. This likely reflected the higher hepatic expression of elovl4, an enzyme that participates in the elongation of ARA and EPA to 22:4n-6 and 22:5n-3, respectively [46], observed in ECO and DCO-fed fish. However, the increased expression of *fads2* was not statistically significant in ECO fed fish, which showed intermediate values between FO and WCO-fed fish. However, increased expression of *fads2* has been reported previously in sea bream fed VO compared to fish fed FO [21, 22]. The involvement of *elovl4* in only later stages of the biosynthetic pathway (predominantly elongation of C_22_), particularly the synthesis of very long chain fatty acids (VLC-FA), explains why sea bream tissue fatty acid compositions largely reflected dietary fatty acid compositions. This was clear in the PCA (NMDS Plot) analysis where all tissues, except brain, grouped according to the feeds, with ECO and DCO clustering in the same group. This indicated that the fatty acid composition of brain was more conserved and less affected by diet than the other tissues, consistent with other studies in the same species [20] and other teleost species [47, 48].

Inclusion of high levels of VO and reduction of FO in feeds has been associated with increased tissue lipid deposition in several fish species [13, 14, 33, 49–51]. In the present study increased lipid deposition was observed only in liver, being highest in WCO-fed fish with ECO and DCO-fed fish showing intermediate values, with liver histology following the same trend. Similar results were found in Atlantic salmon fed high ECO, reflecting the hybrid nature of the GM-derived oil [13, 14]. The mechanism for increased lipid deposition in fish fed VO is not clear although high n-3 LC-PUFA levels found in FO can suppress triacylglycerol (TAG) accumulation in mammalian pre-adipocytes [52] and lipid accumulation in Atlantic salmon adipocytes [53]. Another explanation for the enhanced lipid deposition could be the high levels of both C_18_ fatty acids, 18:2n-6 and 18:3n-3, together with the limited LC-PUFA biosynthetic capacity of this species [22]. In the present study we evaluated the expression of genes involved in lipid and energy metabolism, as well as transcription factors such as SREBP1, which plays a key role in lipid metabolism participating in fatty acid metabolism and *de novo* lipogenesis [54]. Up-regulation in *srebp1* expression in liver was observed in fish fed the three VO in agreement with studies carried out in elovl5^−/−^ mice that indicated low levels of EPA and DHA lead to activation of this transcription factor [55]. Similar results have been obtained in other fish species when fed low levels of n-3 LC-PUFA or VO [50, 56–58]. Up-regulation of *srebp1* in VO-fed fish could lead to the regulation of *srebp1* target genes such as *fads2*, *hl*, *lpl*, *cpt1a* and *cpt1b*. Hydrolysis of TAG in lipoproteins is mediated by *lpl*, and *hl* converts intermediate density lipoprotein to low density lipoprotein, mediating uptake of fatty acids by tissues. Thus, enhanced expression of these two lipase genes would enhance lipid deposition in liver in agreement with a previous study in turbot (*Scophtalmus maximus*) fed soybean oil [50]. On the other hand, *cpt1a*, a gene involved in fatty acid oxidation was up-regulated in fish fed the GM-derived oils and, although it is a *srebp1* target gene, down-regulation of this gene was expected [50]. However, in comparison to the clear effects on liver *cpt* expression in the present study, published data show only marginal effects of replacing FO by VO on β-oxidation [59, 60]. In addition, there are other nutrients that can affect the regulation of these genes such as carbohydrate that can affect lipid metabolism, enhancing the expression of *srebp1* and *cpt1a* and *cpt1b* in rainbow trout (*Oncorhynchus mykiss*) when fed VO [18].

Two other transcription factors, *PPARα* and *PPARγ*, that regulate lipid metabolism and energy homeostasis [61] were evaluated in the present trial, but no regulation of *pparα* was noted in either liver or anterior intestine. PPARα activation by n-3 LC-PUFA may induce the expression of lipolytic genes, such as *cpt*, enhancing fatty acid oxidation [62]. However, in the present study and others, no regulation of *pparα* expression has been observed in fish fed more sustainable diets based on VO [50, 63–65]. In contrast, feeding VO, particularly the GM-derived oils, increased *pparγ* expression. PPARγ activation in mice is known to increase glycolysis, fat storage, fatty acid desaturation and elongation [66]. Thus, up-regulation of *pparγ* may be a compensatory mechanism to attenuate the increased lipid deposition found in fish fed high levels of VO, although it did not have a direct effect on lipolytic genes. Consistent with this, *pparγ* expression was up-regulated in mice fed the high-EPA oil (ECO) compared to mice fed a FO-based diet [67].

Acyltransferases such as LPCAT play a major role in phospholipid remodelling as they alter the fatty acid composition of phospholipids at the *sn*-2 position [68]. The expression of this gene tended to increase in VO-fed fish compared to FO-fed fish in both liver and intestine. This could be reflected in enhanced conversion of lysophosphatidylcholine to phosphatidylcholine in VO-fed fish. Although there is only limited information regarding the regulation of this gene in teleosts, n-3 PUFA have been found to regulate *lcpat1* in larvae of Senegalese sole (*Solea senegalensis*), where gene expression was up-regulated in larvae fed diets containing linseed oil compared to other sources of VO, although no differences were observed with fish fed FO [69]. It is difficult to make comparisons with this study, as fast growing larvae have a high requirement for dietary phospholipids [70] but it is clear that nutritional regulation by n-3 PUFA or LC-PUFA occurs regardless of the life stage. In fact, *lpcat1* regulation has also been found in sea bream [71] and sea bass [72] subjected to fasting, although the direction of regulation varied among species. Also *fabp2*, a gene related to fatty acid transport and uptake, was regulated in liver, being clearly up-regulated in fish fed ECO. Similarly, Atlantic cod (*Gadus morhua*) fed plant meal diets showed a trend towards up-regulation of *fabp2* compared to fish fed fishmeal, in this case associated with lower fat digestibility of the plant-based feeds [73]. Although we cannot fully explain this result at present, we suggest that the up-regulation may be associated with the lower growth of these fish, which in turn could elicit compensatory mechanisms such as fatty acid mobilization.

Some genes were analysed in order to evaluate the immune system and fish health in response to dietary oils. Caspases are central regulators of apoptosis, while PCNA is a marker of cell proliferation, also expressed in non-dividing cells undergoing DNA synthesis and repair [74]. In previous studies, expression of caspases was affected by replacement of dietary FO by VO in fish [63, 64, 75]. Apoptosis is particularly important in tissues with high cell turnover rate such as liver [76] but, in addition, their expression can be altered by factors such as pathological or toxic conditions [77]. Certain VO have been found to enhance oxidative stress and cellular damage [78], and DHA can suppress caspase 3 activation and cell death in neurons [79], which suggests FO diets could have a cellular protective effect in fish. In contrast, the lack of nutritional regulation in anterior intestine was consistent with the results observed in sea bass fed diets containing soybean oil [80]. Expression of *pcna* was not altered when fish were fed the VO-feeds consistent with a previous study in Atlantic salmon [75]. Interleukin 8 (*il8*), a cytokine that serves as a chemical messenger in innate and adaptive immune systems, showed differential regulation in liver and intestine of fish fed VO, probably related to the specific functions of each tissue and their response to nutritional challenge. In this respect, fish gut epithelium acts and reacts as the first line of protection against potentially harmful substances in the diet, with VO causing changes in fish that could favour intestinal dysfunction, including alterations in the gut associated immune system [81]. Thus, down-regulation of *il8* in intestine of VO-fed fish confirmed the importance of dietary fatty acid profile on the immune system of marine fish in agreement with previous studies [80, 82]. In contrast, liver of DCO-fed fish showed higher expression of *il8*, albeit not different to expression in liver of bream fed the other VO diets, which may indicate that the VO fatty acid profile may enhance *il8* activity in this tissue.

In summary, both genetically engineered Camelina oils (EPA only or EPA + DHA) were shown to be viable sources of n-3 LC-PUFA and potential candidates to replace FO in feeds for sea bream, with the growth of fish fed DCO similar to that of fish fed FO. Both oils improved the nutritional quality of the fish fillet, enhancing the n-3 LC-PUFA levels compared to the fish fed the regular (wild-type) VO. Limited LC-PUFA biosynthesis was observed, specifically in liver of fish fed ECO, reflected in higher levels of n-3 DPA, consistent with increased expression of *elovl4* elongase. In general, gene expression reflected the “hybrid” fatty acid composition of the GM-derived oils, eliciting responses that were between the levels of expression in FO and WCO-fed fish, although often more similar to WCO. This may suggest the importance of not only EPA and DHA, but also C_18_ PUFA levels in marine fish lipid metabolism. Based on histology and gene expression, no adverse effects on fish health were observed and thus the cause for the slight reduction in feed intake and consequent fish growth observed in fish fed ECO was not clear but may be related to dietary ARA levels and/or LC-PUFA ratios. Longer term trials with fish up to market size are required to further validate the feasibility of oils from transgenic oilseed crops in marine fish aquaculture.

## Abbreviations

ACTB: β Actin
ADC: Apparent digestibility coefficient
ARA: Arachidonic acid
CASP: Caspase
CPT: Carnitine palmitoyltransferase 1
CYTB: Cytochrome b
DCO: EPA + DHA Camelina oil
DHA: Docosahexaenoic acid
DPA: Docosapentaenoic acid
ECO: EPA-Camelina oil
ELF1α: Elongation factor 1α
ELOVL: Fatty acid elongase
EPA: Eicosapentaenoic acid
FABP2: Fatty acid binding protein 2
FADS2: Fatty acyl desaturase 2
FAME: Fatty acid methyl esters
FCR: Feed conversion ratio
FM: Fish meal
FO: Fish oil
GC: Gas–liquid chromatography
GM: Genetically modified
HL: Hepatic lipase
HSI: Hepatosomatic index
IL: Interleukin
K: Condition factor
LC-PUFA: Long chain polyunsaturated fatty acid(s)
LPCAT: Lysophosphatidylcholine acyltransferase
LPL: Lipoprotein lipase
LTB4: Leukotriene B_4_
MUFA: Monounsaturated fatty acid(s)
n-3: Omega 3
n-6: Omega 6
n.d.: Not determined
NMDS: Non-metric multidimensional scaling plot
NPTII: Kanamycin resistance gene
NTC: Non-template control
PCA: Principal component analysis
PCNA: Proliferating cell nuclear antigen
PGE2: Prostaglandin E_2_
PPARα: Peroxisome proliferator-activated receptor α
PUFA: Polyunsaturated fatty acid(s)
SAFA: Saturated fatty acid(s)
SGR: Specific growth rate
SREBP: Sterol regulatory element binding protein 1
TAG: Triacylglycerol(s)
VLC-FA: Very long chain fatty acid(s)
VO: Vegetable oil(s)
VSI: Viscerosomatic index
WCO: Wild-type Camelina oil
